# Comparative Evaluation of Automated Nucleic Acid Extraction Systems for DNA and RNA Viral Target

**DOI:** 10.3390/pathogens15010071

**Published:** 2026-01-09

**Authors:** Davide Treggiari, Concetta Castilletti, Lavinia Nicolini, Cristina Mazzi, Francesca Perandin, Fabio Formenti

**Affiliations:** 1 Department of Infectious-Tropical Diseases and Microbiology, IRCCS Sacro Cuore Don Calabria Hospital, 37024 Negrar di Valpolicella, Verona, Italy; 2 Clinical Research Unit, IRCCS Sacro Cuore Don Calabria Hospital, 37024 Negrar di Valpolicella, Verona, Italy

**Keywords:** RNA virus, DNA virus, clinical matrix, whole blood, diluted whole blood, plasma, serum, urine, automated nucleic-acid extraction system, silica column-based, magnetic bead-based

## Abstract

Background: Efficient nucleic acid extraction is essential for reliable viral load testing, yet performance can differ widely depending on the extraction system and sample type. We compared three automated platforms, QIAcube, EZ1 Advanced, and Maxwell RSC, for their ability to recover cytomegalovirus (CMV) DNA and West Nile virus (WNV) RNA from common clinical matrices. Methods: Mock specimens were prepared using whole blood, plasma, serum, and urine collected from two donors. Samples were spiked with CMV or WNV culture material and extracted in triplicate on each platform according to the manufacturers’ protocols. Viral loads were measured using ELITech ELITE MGB assays on the InGenius system. Whole blood samples were also tested after a 1:4 dilution. Matrix-specific standard curves were applied, and viral loads were compared using Wilcoxon rank-sum tests with false-discovery rate adjustment. Results: Extraction efficiency differed substantially by platform and specimen type. For CMV, QIAcube consistently produced the highest DNA recovery across all matrices, with particularly large differences in plasma and serum, where EZ1 and Maxwell RSC yielded significantly lower loads. The WNV results varied by matrix: EZ1 and QIAcube performed similarly in plasma, while Maxwell RSC achieved the highest RNA recovery in whole blood. While the QIAcube exhibited reduced WNV recovery in blood, it achieved the best performance in serum, as specified by the kit. No significant platform differences were observed for urine. Diluting whole blood reduced variability between platforms. Conclusions: Both sample matrix and extraction system strongly influence nucleic acid recovery. These results highlight the need for matrix-specific validation and standardized extraction approaches in molecular diagnostics.

## 1. Introduction

Efficient and reproducible extraction of nucleic acids represents a critical prerequisite for all molecular diagnostic assays [1,2]. Regardless of the amplification technology employed, the analytical sensitivity, quantitative accuracy, and inter-laboratory comparability of molecular tests largely depend on the quality, purity, and recovery efficiency of the extracted DNA or RNA [3]. Variability introduced at the extraction step is a well-recognized source of measurement bias in viral load testing and remains one of the major obstacles to full standardization of molecular diagnostics [4,5].

Over the past two decades, automated nucleic acid extraction platforms have progressively replaced manual methods in routine diagnostic laboratories. Automation offers several advantages, including reduced hands-on time, improved biosafety, higher throughput, and increased reproducibility [6,7]. However, performance differences between automated systems persist due to variations in extraction chemistry (silica columns vs. magnetic beads), lysis conditions, wash stringency, and elution strategies. These differences can directly affect nucleic acid yield, purity, and downstream amplification efficiency, with measurable consequences on nucleic acid quantification and clinical interpretation [8,9].

The biological matrix represents one of the most important determinants of extraction performance. Whole blood, plasma, serum, and urine differ markedly in cellular content, protein composition, enzyme activity, and the presence of endogenous substances that may inhibit nucleic acid amplification [10]. Whole blood, in particular, contains high concentrations of heme, immunoglobulins, and cellular debris that can interfere with both extraction efficiency and PCR performance [7]. Urine is characterized by variable pH and nuclease activity that can compromise RNA stability. As a consequence, an extraction method that performs optimally in one biological matrix may show significantly reduced efficiency in another [11,12].

The type of nucleic acid further influences recovery. RNA is inherently more labile than DNA and is more susceptible to degradation during pre-analytical handling and extraction. Furthermore, some extraction chemistries may be optimized for DNA recovery but may perform sub-optimally for RNA, particularly in complex matrices such as whole blood [13,14]. Despite the clinical impact of these issues, comparative evaluations that systematically assess extraction efficiency across both DNA and RNA targets and across multiple biological matrices remain relatively limited.

In this context, viral model targets provide a practical and controlled strategy for studying extraction performance. DNA viruses and RNA viruses differ fundamentally in genome structure and stability, allowing for the investigation of extraction-related biases linked to nucleic acid type. Cytomegalovirus (CMV) and West Nile virus (WNV) represent well-established laboratory targets for DNA and RNA, respectively. CMV is widely used as a benchmark for quantitative viral diagnostics and inter-laboratory harmonization [15,16], while WNV represents a clinically relevant RNA virus requiring high analytical sensitivity, particularly in the context of blood and organ donation screening [17,18].

The aim of the present study was to perform a comparative analytical evaluation of three widely used automated nucleic acid extraction systems—QIAcube (silica column-based), EZ1 Advanced (magnetic bead-based), and Maxwell RSC (magnetic bead-based)—using standardized spiked samples. Extraction performance was assessed for both a DNA viral target (CMV) and an RNA viral target (WNV) across four different biological matrices: whole blood, plasma, serum, and urine. In addition, spiked whole blood samples were subsequently diluted 1:4 and subjected to the same extraction processes. By controlling pre-analytical variables and using a single real-time PCR platform for quantification, this study specifically addresses the impact of extraction platform, nucleic acid type, and biological matrix on viral nucleic acid recovery, with the objective of providing practical methodological guidance for laboratories implementing or validating automated extraction workflows.

## 2. Materials and Methods

### 2.1. Study Design

This study compared the analytical performance of three automated nucleic acid extraction platforms. QIAcube (QIAamp Viral RNA Kit and QIAamp DNA Blood Kit, Qiagen, Hilden, Germany), EZ1 Advanced (DSP Virus Kit, Qiagen, Germany), and Maxwell RSC (RSC Pathogen total nucleic acid kit, Promega, Madison, WI, USA). Two independent donors were used to introduce biological variability, and four biological matrices were tested: whole blood, plasma, serum, and urine. Two experimental batches were prepared, spiked with CMV or WNV viral stocks. In addition, spiked diluted whole blood samples were also used.

### 2.2. Virus Stock Preparation

Vero E6 (American Type Culture Collection (ATCC), CRL-1586, Manassas, VA, USA) and MRC-5 (ATCC, CCL-171) cells were both maintained in Eagle’s Minimum Essential Medium (EMEM) (Gibco, Thermo Fisher Scientific Inc., Walthaman, MA, USA) supplemented with 100 U/mL penicillin/streptomycin (Gibco) and 10% heat-inactivated fetal calf serum (FCS) (Gibco) at 37 °C and 5% CO_2_ in a humidified atmosphere. For WNV stock preparation, Vero E6 cells were infected with WNV Lineage 2 (kindly provided by Professor Tiziana Lazzarotto, Regional Reference Centre for Microbiological Emergencies (CRREM), St. Orsola Malpighi University Hospital, University of Bologna, Bologna, Italy) under biosafety level (BSL)-3 conditions, incubated at 37 °C and 5% CO_2_ in a humidified atmosphere and followed to 90% cytopathic effect (CPE) development. Cell lysates were then clarified, aliquoted, and stored at −80 °C until use. Virus titration was performed on the Vero E6 cell line using a limiting dilution assay; the titer was calculated using the method of Reed and Muench and expressed as the tissue culture infectious dose TCID_50_/mL WNV stock titer was 10^8^ TCID_50_/mL.

MRC-5 cells were infected with CMV (ATCC^®^ number VR-538, strain AD-169) that was propagated on MRC-5 cells under BSL-2 conditions as described above. CMV stock titer was 10^6.75^ TCID_50_/mL. Both viral preparations were than tested with ELITech CMV and WNV ELITE MGB kit (Turin, Italy), respectively, on the InGenius (package version 1.3.0.19) platform to have information on Ct values and copies/mL (CMV: Ct 27.37, 257,691 copies/mL; WNV: Ct 21.16, 42,528,641 copies/mL).

### 2.3. Sample Preparation and Viral Spiking

Whole blood was collected from two healthy volunteer donors in EDTA tubes and processed separately. The collected whole blood was spiked with CMV or WNV cell cultures supernatants to target a cycling threshold (Ct) of 30 ± 2. Spiked whole blood samples were subsequently diluted 1:4 (1:4 (*v*:*v*) in a phosphate-buffered solution (PBS, pH 7.4, Gibco). Aliquots of 200 µL were prepared from the spiked whole blood and stored at −20 °C for subsequent analysis. The remaining spiked whole blood was centrifuged at 1976 RCF for 10 min to obtain plasma, which was then aliquoted (200 µL) and stored at −20 °C. Urine and serum samples were collected from the same donors, processed similarly, and spiked with the same viral dilutions. All matrices were prepared using the same protocol for both donors.

### 2.4. Nucleic Acid Extraction

Each matrix was extracted in triplicate on all three extraction platforms following the manufacturers’ protocols. EZ1 (DSP Virus Kit, Qiagen, Germany) and Maxwell (RSC Pathogen total nucleic acid kit, Promega, USA) systems used magnetic-bead-based extraction, whereas QIAcube (QIAamp Viral RNA Kit and QIAamp DNA Blood Kit, Qiagen, Germany) performed silica spin-column-based purification. The QIAamp Viral RNA Kit is validated for the extraction of viral RNA from plasma, serum, urine, while the QIAamp DNA Blood Kit is validated for purification of DNA from whole blood, plasma, serum. The EZ1 DSP Virus Kit is validated for automated extraction of viral nucleic acids from plasma, serum, and other cell-free body fluids, and the Maxwell RSC Pathogen Total Nucleic Acid Kit is validated for simultaneous purification of viral DNA and RNA from plasma, serum, whole blood, urine. Input and elution volumes differed across instruments; for CMV, 200 µL input and 100 µL elution were used on QIAcube, and Maxwell, while EZ1 used 200 µL input and 90 µL elution. For WNV, QIAcube processed 140 µL input with 65 µL elution; EZ1 and Maxwell used 200 µL input and 90 µL elution. All eluates were stored at −20 °C until PCR.

### 2.5. Amplification and Quantification

Eluates were tested in duplicate using ELITech CMV and WNV ELITE MGB kits on the InGenius platform (Qiagen, Germany). Ct values and copies/mL were automatically generated. Because the extraction volumes varied, the final viral load results were then corrected for differences in starting sample volume and elution volume. This volume-adjusted approach minimized bias arising from different concentration factors among the extraction platforms.

### 2.6. Statistical Analysis

Continuous variables were expressed as median and interquartile range (Q1–Q3) or as mean and standard deviation (SD), as appropriate, based on data distribution assessed through graphical methods and normality tests. For each biological matrix, the viral load (copies/mL, calculated from the standard curve) was summarized as the median and interquartile range (Q1–Q3). Due to the non-normal distribution of the data, non-parametric statistical tests were applied. Pairwise comparisons between extractors (EZ1 vs. Maxwell, EZ1 vs. QIAcube, Maxwell vs. QIACube) were performed using the Wilcoxon rank-sum test with false discovery rate (FDR) correction for multiple testing. These analyses were performed for both targets (CMV and WNV) independently.

All statistical analyses were performed using R statistical software (version 4.5.1), and significance was set at *p* < 0.05 after correction for multiple comparisons.

## 3. Results

### 3.1. CMV

The comparative evaluation of the three automated extraction platforms revealed marked differences in CMV DNA recovery depending on the biological matrix (Table 1).

Plasma: QIAcube extraction system produced the highest median CMV DNA yield (82,972 copies/mL), which was significantly higher than that of both EZ1 and Maxwell RSC (33,560 copies/mL and 15,633 copies/mL, respectively; *p* < 0.001 for both). 

Whole blood: Maxwell RSC showed the highest CMV DNA recovery (median 118,577 copies/mL), significantly outperforming EZ1 (23,589 copies/mL; *p* < 0.001), while no statistically significant difference was observed between Maxwell RSC and QIAcube (*p* = 0.436). Maxwell RSC is validated for whole blood, which may contribute to its higher efficiency in this matrix.

Diluted whole blood: Both QIAcube (51,293 copies/mL) and Maxwell RSC (43,224 copies/mL) yielded significantly higher CMV DNA amounts than EZ1 (7409 copies/mL; *p* < 0.001). Although Maxwell RSC is validated for whole blood, the difference between Maxwell RSC and QIAcube was not statistically significant (*p* = 0.069).

Urine: No significant difference was observed between EZ1 and Maxwell RSC (*p* = 0.751). However, QIAcube showed the highest CMV DNA recovery (143,477 copies/mL), significantly outperforming both magnetic-bead-based systems (*p* < 0.001). EZ1 and Maxwell RSC showed comparable results, but their kits are validated for plasma/serum or cell-free fluids rather than urine, which may partially explain the lower recovery.

Serum: QIAcube again provided the highest CMV DNA yield (239,658 copies/mL), followed by Maxwell RSC (60,199 copies/mL) and EZ1 (36,558 copies/mL). All pairwise comparisons were statistically significant (*p* = 0.009).

Overall, these results indicate differences in performance among the extraction systems, with the QIAcube extraction system showing favorable performance in cell-free matrices such as plasma, serum, and urine (low-cell-content matrices), while the Maxwell RSC showed better performance in whole-blood specimens.

### 3.2. WNV

Extraction performance for WNV RNA also showed pronounced matrix-dependent variability among platforms (Table 2).

Plasma: EZ1 (41,606 copies/mL) and QIAcube (41,625 copies/mL) yielded comparable WNV RNA concentrations and both significantly outperformed Maxwell RSC (25,698 copies/mL; *p* = 0.018). No significant difference was observed between EZ1 and QIAcube (*p* = 0.544).

Whole blood: Maxwell RSC (6562 copies/mL) and EZ1 (5695 copies/mL) yielded significantly higher WNV RNA amounts than QIAcube (247 copies/mL; *p* < 0.001 for both comparisons). No significant difference was observed between Maxwell RSC and EZ1 (*p* = 0.112). QIAcube RNA kit is not validated for whole blood, which likely explains the markedly lower recovery.

Diluted whole blood: Maxwell RSC showed the highest WNV RNA recovery (10,669 copies/mL), significantly outperforming both EZ1 (2115 copies/mL; *p* = 0.001) and QIAcube (558 copies/mL; *p* = 0.040).

Urine: No statistically significant differences in WNV RNA recovery were observed among the three extraction platforms in urine (*p* = 0.885).

Serum: QIAcube produced the highest WNV RNA yield (37,785 copies/mL), followed by Maxwell RSC (28,086 copies/mL) and EZ1 (24,623 copies/mL). All pairwise comparisons were statistically significant (*p* = 0.035).

Collectively, these findings indicated that EZ1 and Maxwell RSC were more efficient for WNV RNA recovery in blood-based matrices, whereas QIAcube performed best in serum.

## 4. Discussion

This comparative evaluation demonstrates that both the biological matrix and the extraction platform substantially influence nucleic acid recovery for both DNA and RNA viral targets. For CMV, the most pronounced differences were observed in plasma, serum, and urine, where QIAcube consistently showed superior recovery and reproducibility. It should be noted that QIAcube employed a DNA-specific extraction kit (QIAamp DNA Blood Kit), whereas EZ1 and Maxwell RSC used total nucleic acid kits, which likely contributed to the higher CMV DNA yields observed with QIAcube in liquid matrices. These findings are in agreement with previous investigations showing that extraction chemistry and matrix composition significantly affect CMV DNA quantification and contribute to inter-laboratory variability [5,19].

For WNV RNA, QIAcube showed the highest recovery in serum, while EZ1 and Maxwell demonstrated superior performance in whole blood matrices. In this case, although a specific RNA kit (QIAamp Viral RNA Kit) was used, this was only suitable for cell-free matrices, which could explain the higher RNA recovery observed in serum and lower recovery in blood.

Interestingly, the marked reduction in WNV RNA recovery observed with QIAcube in whole blood might likely reflect virus-specific and nucleic-acid-specific extraction efficiency. WNV is a single-stranded RNA virus, which is inherently more labile than double-stranded DNA and is therefore more susceptible to degradation during pre-analytical handling and extraction. This instability is particularly critical in complex matrices such as whole blood, where endogenous RNases and heme-derived compounds can compromise RNA integrity [7,20]. In contrast, CMV is a large double-stranded DNA virus and is more robust during extraction, resulting in more consistent recovery across platforms.

Several methodological limitations should be acknowledged. First, although multiple biological matrices were evaluated to explore matrix-related variability, plasma remains the clinically recommended specimen for CMV viral load monitoring. International diagnostic guidelines consistently emphasize plasma as the most reliable compartment for CMV DNA quantification in transplant recipients and immunocompromised patients [15,16]. Therefore, while the analysis of alternative matrices provides valuable methodological insights, their direct clinical interpretability remains limited.

Second, only two biological donors were included in this study. While this design minimized biological variability and allowed for the assessment of technical repeatability, it does not fully represent the heterogeneity encountered in clinical specimens, such as variations in hematocrit, leukocyte content, protein concentration, or endogenous PCR inhibitors. All of these factors are known to influence extraction efficiency and amplification performance [7,21]. Future studies including a larger number of donors and true clinical samples will be required to confirm the generalizability of these findings.

Third, all amplification and quantification steps were performed using the InGenius platform. Although this approach minimized inter-platform PCR variability, the use of a single manufacturer’s system for amplification may introduce a systematic analytical bias [6]. Independent quantification on an external real-time PCR platform would reduce this potential source of bias. Furthermore, despite the application of a volume-adjusted normalization strategy, differences in extraction input and elution volumes may still contribute to residual variability in apparent viral load recovery, as previously reported in inter-platform standardization studies [4].

Finally, the use of laboratory-spiked samples does not fully replicate the biological complexity of naturally infected clinical specimens. Artificial spiking preserves viral integrity and concentration but does not reflect the heterogeneous viral distribution, nucleic acid degradation, or protein–virus interactions observed in vivo [22]. In CMV infection specifically, viral DNA can be present in both plasma and leukocyte-associated compartments, which may exhibit distinct recovery efficiencies depending on extraction chemistry [23].

Overall, this study provides an important methodological benchmark for laboratories evaluating automated nucleic acid extraction strategies for viral diagnostics. The data clearly demonstrate that extraction performance is strongly dependent on both the biological matrix and the viral nucleic acid type. The observed differences between QIAcube and the magnetic-bead-based platforms highlight the impact of kit design (DNA specific vs. total nucleic acid) on extraction efficiency, particularly for DNA viruses. Additional validation using true clinical specimens will be essential to translate these findings into standardized diagnostic practice and to support harmonized inter-laboratory viral load comparisons.

## 5. Conclusions

This study compares three commonly used automated nucleic acid extraction systems across different sample types and for both DNA and RNA viruses. The findings show that extraction performance depends strongly on the specimen matrix and the type of nucleic acid, with clear differences between platforms. QIAcube performed best for CMV DNA, especially in plasma, serum, and urine. Maxwell RSC showed superior recovery of WNV RNA from whole blood. EZ1 generally showed moderate or lower performance, varying by matrix. RNA extraction was particularly affected by the extraction method in complex samples, underscoring the greater fragility of RNA during pre-analytical steps. Overall, the results demonstrate that extraction platforms are not interchangeable and must be validated for specific targets and sample types. This work offers practical guidance for laboratories selecting extraction systems and highlights the importance of standardized workflows to improve consistency between molecular diagnostic results.

## Figures and Tables

**Table 1:** Comparison of viral CMV DNA recovery across different biological matrices and extraction platforms. Viral DNA was measured using real-time PCR. Each sample was extracted in triplicate and tested in duplicate. The results are reported as median copies/mL (Q1–Q3 in parentheses). Statistical comparisons were performed using the Wilcoxon rank-sum test with FDR correction (PCR performed on the ELITe InGenius system). * Diluted whole blood sample consisting of virus spiked whole blood sample subsequently diluted 1:4 (*v*:*v*) in PBS.

Biological Matrix	Extractor			Multiple Comparisons *p* Value		
	EZ1	Maxwell	QIAcube	EZ1 vs. Maxwell	EZ1 vs. QIAcube	Maxwell vs. QIAcube
	*n* = 12	*n* = 12	*n* = 12			
Plasma	33,560.33 (31,163.85–35,483.85)	15,633.00 (10,514.00–21,771.50)	82,971.50 (73,133.00–115,715.00)	0.001	0.000	0.000
Whole blood	23,589.00 (19,470.60–28,926.23)	118,576.50 (62,204.50–137,976.50)	81,852.50 (75,048.00–116,729.00)	0.000	0.000	0.436
Diluted whole blood *	7409.03 (6638.85–7962.98)	43,223.50 (35,068.50–47,151.00)	51,293.00 (43,820.00–58,789.00)	0.000	0.000	0.069
urine	57,974.40 (43,727.40–81,262.13)	61,436.50 (51,635.50–82,279.00)	143,476.50 (92,037.00–156,139.00)	0.751	0.000	0.000
serum	36,557.55 (28,102.28–46,527.53)	60,198.50 (50,924.50–65,021.00)	239,658.00 (214,048.50–264,635.00)	0.009	0.004	0.000

**Table 2:** Comparison of viral WNV RNA recovery across different biological matrices and extraction platforms. Viral RNA was quantified using real-time PCR. Each sample was extracted in triplicate and analyzed in duplicate. The results are reported as median copies/mL (Q1–Q3). Statistical comparisons were conducted using the Wilcoxon rank-sum test with FDR correction, (PCR performed on the ELITe InGenius system). * Diluted whole blood sample consisting of virus spiked whole blood sample subsequently diluted 1:4 (*v*:*v*) in PBS.

Biological Matrix	Extractor			Multiple Comparisons *p* Value		
	EZ1	Maxwell	QIAcube	EZ1 vs. Maxwell	EZ1 vs. QIAcube	Maxwell vs. QIAcube
	*n* = 12	*n* = 12	*n* = 12			
Plasma	41,606.33 (36,783.68–49,245.08)	25,697.50 (21,262.00–38,937.50)	41,625.07 (35,940.13–45,097.93)	0.018	0.544	0.018
Whole blood	5695.43 (4702.50–6454.35)	6562.00 (5565.00–7403.50)	247.43 (240.50–383.04)	0.112	0.000	0.000
Diluted whole blood *	2115.23 (1134.90–5217.30)	10,668.50 (9701.50–12,101.00)	557.61 (424.82–1214.57)	0.001	0.040	0.001
urine	20,818.80 (8670.38–38,522.03)	21,339.50 (7566.50–55,904.50)	13,528.13 (9207.02–37,371.75)	0.885	0.885	0.885
serum	24,623.10 (22,717.58–26,130.83)	28,085.50 (26,807.50–33,407.50)	37,784.50 (32,266.93–40,811.41)	0.002	0.000	0.035

## Data Availability

The original contributions presented in this study can be directed requested to the corresponding author.
